# Using e-health in perioperative care: a survey study investigating shortcomings in current perioperative care and possible future solutions

**DOI:** 10.1186/s12893-017-0254-6

**Published:** 2017-05-23

**Authors:** Eva van der Meij, Esther V.A. Bouwsma, Baukje van den Heuvel, H. Jaap Bonjer, Johannes R. Anema, Judith A.F. Huirne

**Affiliations:** 1 Department of Public and Occupational Health, EMGO+ Institute for Health and Care Research, VU University Medical Center, van der Boechorststraat 7, 1081 BT Amsterdam, The Netherlands; 2 Department of Obstetrics and Gynaecology, VU University Medical Center, Amsterdam, The Netherlands; 3 Department of Surgery, Jeroen Bosch Ziekenhuis, Den Bosch, The Netherlands; 4 Department of Surgery, VU University Medical Center, Amsterdam, The Netherlands

**Keywords:** E-health, Recovery, Perioperative care, Abdominal surgery, Hernia inguinal surgery, Cholecystectomy, Appendectomy, Colectomy, Adnexal surgery, Hysterectomy

## Abstract

**Background:**

An e-health care program has previously shown to have a positive effect on return to work, quality of life and pain in patients who underwent gynaecological surgery. Plausibly, providing the care program to a population undergoing other types of surgery will be beneficial as well. The objectives of this study are to evaluate patients’ opinions, needs and preferences regarding the information and guidance supplied to patients during the perioperative period, to investigate whether e-health may be of assistance and to explore if gender specific needs exist.

**Methods:**

A questionnaire was sent to all patients between 18 and 75 years (*n* = 362), who underwent various forms of abdominal surgery between August 2013 to September 2014 in a university hospital in the Netherlands. The questionnaire contained questions about the current situation in perioperative care and questions about patients’ preferences in an e-health care program. Gender differences were evaluated.

**Results:**

Two hundred seven participants (57.2%) completed the survey. The majority of the participants were relatively satisfied with the perioperative care they received (68.6%). Most reported shortcomings in perioperative care concerning the supply of information regarding the resumption of activities and guidance during the recovery course. An e-health care program was expected to be of added value in perioperative care by 78% of the participants; a website was reported as most useful. In particular practical functions on a website focusing on the preparation to surgery and monitoring after surgery were appraised to be highly valuable. Overall, women had slightly more needs for extra information and support during the perioperative course than men.

**Conclusions:**

In abdominal surgery, there is a need for an e-health care program, which should focus mainly on the supply of information about the resumption of activities as well as guidance in the postoperative course.

## Background

Postoperative recovery often takes much longer than the period considered appropriate by specialists [1–5]. An important predictor for the length of recovery is the level of invasiveness of the surgical procedure. In addition, patient expectations about their recovery influence the length of recovery considerably [2, 5, 6]. For this reason, a perioperative e-health intervention focusing on the supply of information with respect to the recovery period after gynecological surgery, was developed in 2011 by a qualitative study using an intervention mapping protocol [7]. Intervention mapping is a systematic description of a logical planning process in several steps, starting with a needs assessment and ending with an evaluation of the developed intervention [8]. The e-health intervention which was developed included an interactive website containing tailored, structured and detailed instructions concerning the resumption of activities after surgery. The effectivity of this intervention was evaluated by a randomized controlled trial; patients who received the e-health intervention in addition to usual perioperative care returned to work nine days earlier compared to the patients who received usual perioperative care only [9]. The care program also had a positive influence on quality of life and perception of pain after 26 weeks.

Plausibly, providing the care program to a population undergoing other types of surgery will be beneficial as well. However, it should be investigated whether the intervention should be adjusted to a new patient population. In addition, the care program was developed five years ago and patients’ needs and preferences nowadays may have changed. Moreover, the e-health intervention was originally developed for female patients undergoing gynecological surgery. It has already been proven that, besides disease specific and biochemical differences, women and men differ on various aspects according to their needs and health care use, requiring additional research on this topic taking gender differences into account [10–16].

In conclusion, patients’ views on perioperative care and their preferences regarding e-health need to be investigated across a broader population, before the earlier developed e-health intervention for gynaecological patients can be offered to all patients undergoing abdominal surgery. Therefor a survey questionnaire was developed for patients who underwent various forms of abdominal surgery. With this study we aim 1) to evaluate shortcomings in the information and guidance supplied to patients in current perioperative care, and 2) to investigate whether e-health may be of assistance in this, and finally 3) if gender specific needs exist.

## Methods

### Study design

A survey questionnaire study was conducted in accordance with the STROBE statement [17]. The medical ethics committee of the VU medical center approved the protocol in 2014 (registration number 2014.378).

### Development of the questionnaire

A questionnaire was developed for this study and was based on the results of a qualitative study which was performed in 2011 to develop the e-health intervention for patients undergoing gynecological surgery [7]. In this study an intervention mapping protocol was used, including a literature search, focus group discussions with patients and questionnaires for patients, medical doctors and e-health specialists. The questionnaire of the present study consisted of two parts. First, gaps in current perioperative care were evaluated and patients’ needs and preferences were investigated. Topics included patients’ mental health state before and after surgery, the information patients received before and after surgery and the guidance and monitoring provided to them during the recovery process. The questions were based on the outcomes of the needs assessment part of the intervention mapping protocol. The second part of the questionnaire consisted of questions about patients’ needs regarding various forms of e-health in perioperative care. These questions were based on the outcomes of the part of the intervention mapping protocol called “the program plan; design of the intervention”. In addition, some questions were added based on the comments of patients who had used the earlier developed e-health intervention in a randomized controlled trial and on additional literature findings [9, 18–21].

### Study population

All patients between 18 and 70 years old who underwent a cholecystectomy, inguinal hernia surgery, appendectomy, colectomy, a hysterectomy or adnexal surgery (all laparoscopic or open), between august 2013 and august 2014 in the VU University Medical Center in Amsterdam, the Netherlands, received an invitation to complete the questionnaire. The surgical procedures were selected as these are the most commonly performed general abdominal surgical and gynecological procedures (apart from Caesarean Section) in the Netherlands [22, 23].

### Data collection

In October 2014, the potential participants received an envelope containing information about the study, the questionnaire and a return envelope. In case patients did not wish to participate they could indicate this by returning a return slip. When the researchers had not received the return slip or the completed questionnaire after 3 or 6 weeks respectively, the participant received a reminder.

Questions with five answering options (for example: really useful, useful, neutral, not useful, not useful at all) were recoded to three answering options, by combining ‘really useful and useful’ and ‘not useful and not useful at all’, to give a clearer overview of the results. Baseline characteristics such as the American Society of Anesthesiologists (ASA) classification, Body Mass Index (BMI), indication for surgery and complications during or after surgery, were collected by screening the medical records of the participants. The level of invasiveness of the surgical procedure was defined as ‘minor surgery’ or ‘other’. Procedures which were defined as minor surgery were laparoscopic cholecystectomy, hernia inguinal surgery (open and laparoscopic), laparoscopic appendectomy or laparoscopic adnexal surgery. This was based on the fact that these types of procedures are related to more or less the same convalescence recommendations after surgery [24, 25]. The remaining procedures were defined as ‘other’ because it was not possible to categorize them into groups because of their heterogeneity according to level of invasiveness.

### Statistical analyses

All statistical analyses were carried out using SPSS version 20.0. Descriptive statistics were used to present the baseline characteristics and responses of the participants. We used cross-tabulations, Chi2-tests and t-tests to compare baseline characteristics between responders and non-responders. Responses were compared according to gender, only in the group of patients who underwent a general surgical procedure with a minor level of invasiveness (laparoscopic cholecystectomy, laparoscopic or open hernia inguinal surgery, laparoscopic appendectomy). Reason for this was to develop the maximum homogeneous group, to limit the effect of potential confounding factors.

## Results

### Response

A total of 362 potential participants were identified and received an invitation to participate. The questionnaire was completed by 207 participants (57.2%). Of 6 potential participants, we were sure that we did not reach them, because the questionnaires were returned to us with the notification that the potential participant had moved. Seventeen potential participants indicated that they were not willing to participate by sending back the return slip and four potential participants were excluded because of a language barrier or cognitive impairment. We performed a comparison of the participants and non-participants regarding some important baseline characteristics (Table 1). This analysis only showed significant differences between responders and non-responders according to age (participants were older than non-participants) and type of surgery (patients who underwent a gynecological procedure were more likely to respond than patients who underwent general surgical procedures). There were no statistically or clinically relevant differences in the health related characteristics which we analyzed. Median time between surgery and the moment of sending the questionnaire to the participants was 38 weeks (range 5–62 weeks).

**Table 1:** Comparison of participants and non-participants

Variable	Participants (*n* = 207)	Non-participants (*n* = 155)	*P*-value
Gender			
Male	56 (27.1%)	47 (30.2%)	0.50
Female	151 (72.9%)	108 (69.7%)	
Age			
(mean, sd)	46.59 (13.39)	39.57 (12.52)	**0.00**
SES			
(mean, sd)	0.64 (1.05)	0.64 (1.18)	0.53
BMI			
*n* = 340	*n* = 200	*n* = 140	0.89
(mean, sd)	27.43 (15.12)	27.78 (18.12)	
ASA classification			
*n* = 279	*n* = 171	*n* = 108	0.53
ASA 1	80 (46.8%)	58 (53.7%)	
ASA 2	82 (48.0%)	39 (36.1%)	
ASA 3	7 (4.1%)	10 (9.3%)	
ASA 4	2 (1.2%)	1 (0.9%)	
Intoxications†			
*n* = 322	*n* = 194	*n* = 128	0.26
Yes	105 (54.1%)	64 (47.8%)	
No	89 (45.9%)	70 (52.2%)	
Type of surgery			
Gynecological	107 (51.7%)	60 (38.7%)	**0.01**
Surgical	100 (48.3%)	95 (61.3%)	
Major complications during or after surgery^a^			
Yes	9 (4.3%)	7 (4.5%)	0.94
No	198 (95.7%)	148 (95.5%)	

Data are presented as frequencies and percentages, unless otherwise stated. The values that differ significantly are highlighted in bold

*SD* standard deviation, *SES* Social Economic Status, Scores are based on geographic location, *BMI* Body Mass Index, *ASA* American Society of Anesthesiologists classification

† Defined as: Any current use of alcohol, tobacco and/or drugs

^a^Defined as: Conversion to an open procedure, re-surgery within 30 days, injury of the bladder, intestine or liver during surgery, or drainage of an abscess after surgery

### Baseline characteristics

Table 2 presents the baseline characteristics of the participants who completed the questionnaire. Most participants were female (*n* = 151, 72.9%) and the indication for surgery was in the majority of the participants benign (*n* = 181, 87.4%). Mean age was 46.6 years. Of the participants, 95.1% used the Internet on a daily base. The subgroup of participants which was used to compare the results of men and women with each other (i.e. who underwent a general surgical procedure with a minor level of invasiveness), consisted of 71 participants (male *n* = 42, female *n* = 29). Men underwent laparoscopic hernia inguinal surgery more often in comparison to women (*n* = 15, 35% vs *n* = 2, 6.9%) and women underwent a laparoscopic cholecystectomy more often compared with men (*n* = 19, 65.5% vs *n* = 12, 26.6%). In addition, age differed remarkably between men and women in this subgroup (52.67 (SD 13.8) vs 41.66 (SD 13.9)) which is possibly due to the difference in surgical procedures. No other clinically differences were found within this subgroup.

**Table 2:** Baseline characteristics

Variable	Total *n* = 207
Gender	
Male	56 (27.1%)
Female	51 (72.9%)
Age (mean sd)	46.59 (13.4)
Nationality	
Dutch	190 (91.8%)
Other	17 (8.2%)
Level of education	
Low	25 (12.1%)
Medium	66 (31.9%)
High	116 (56.0%)
Employment status	
Employed	142 (68.6%)
Non-employed	65 (31.4%)
Internet use	*n* = 203
Daily or more times a week	193 (95.1%)
Seldom or never	10 (4.9%)
Source of Internet use	*n* = 193
Computer/laptop	25 (13.0%)
Smartphone/tablet	38 (19.7%)
Both	130 (67.4%)
BMI	*n* = 200
(mean sd)	26.4 (5.6)
ASA classification	*n* = 171
ASA 1	80 (46.8%)
ASA 2	82 (48.0%)
ASA 3	7 (3.4%)
ASA 4	2 (1.2%)
Type of surgery	
Gynecological	107 (51.7%)
Surgical	100 (48.3%)
Indication for surgery	
Malignancy	26 (12.6%)
Benign	181 (87.4%)
Type of surgery	
Minor^a^	132 (63.8%)
• Adnexal surgery (LS)	61
• Cholecystectomy (LS)	31
• Hernia inguinal surgery (LS)	17
• Hernia inguinal surgery (O)	3
• Appendectomy (LS)	20
Other	75 (36.2%)
• Adnexal surgery (O)	5
• Cholecystectomy (LS)	4
• Appendectomy (LS)	6
• Colectomy (LS)	9
• Colectomy (O)	10
• Hysterectomy (LS)	36
• Hysterectomy (O)	5
Major complications during or after surgery^b^	9 (4.3%)

Data are presented as frequencies and percentages, unless otherwise stated

*BMI* Body Mass Index, *ASA* American Society of Anesthesiologists classification, *LS* laparoscopic procedure, *O* Open procedure

^a^This subdivision is based on a classification which has been used previously in gynaecologic surgery [1, 5]. The general surgical procedures were classified in line with this classification, based on the length of convalescence recommendations for resumption of activities after these general surgical and gynaecological procedures. These convalescence recommendations were developed in a Delphi study [24, 25]

^b^Defined as: Conversion to an open procedure, re-surgery within 30 days, injury of the bladder, intestine or liver during surgery, or drainage of an abscess after surgery

### Patients’ views on the available information and provided guidance in perioperative care

#### Before surgery

##### Mental health state

**Fig. 1:**
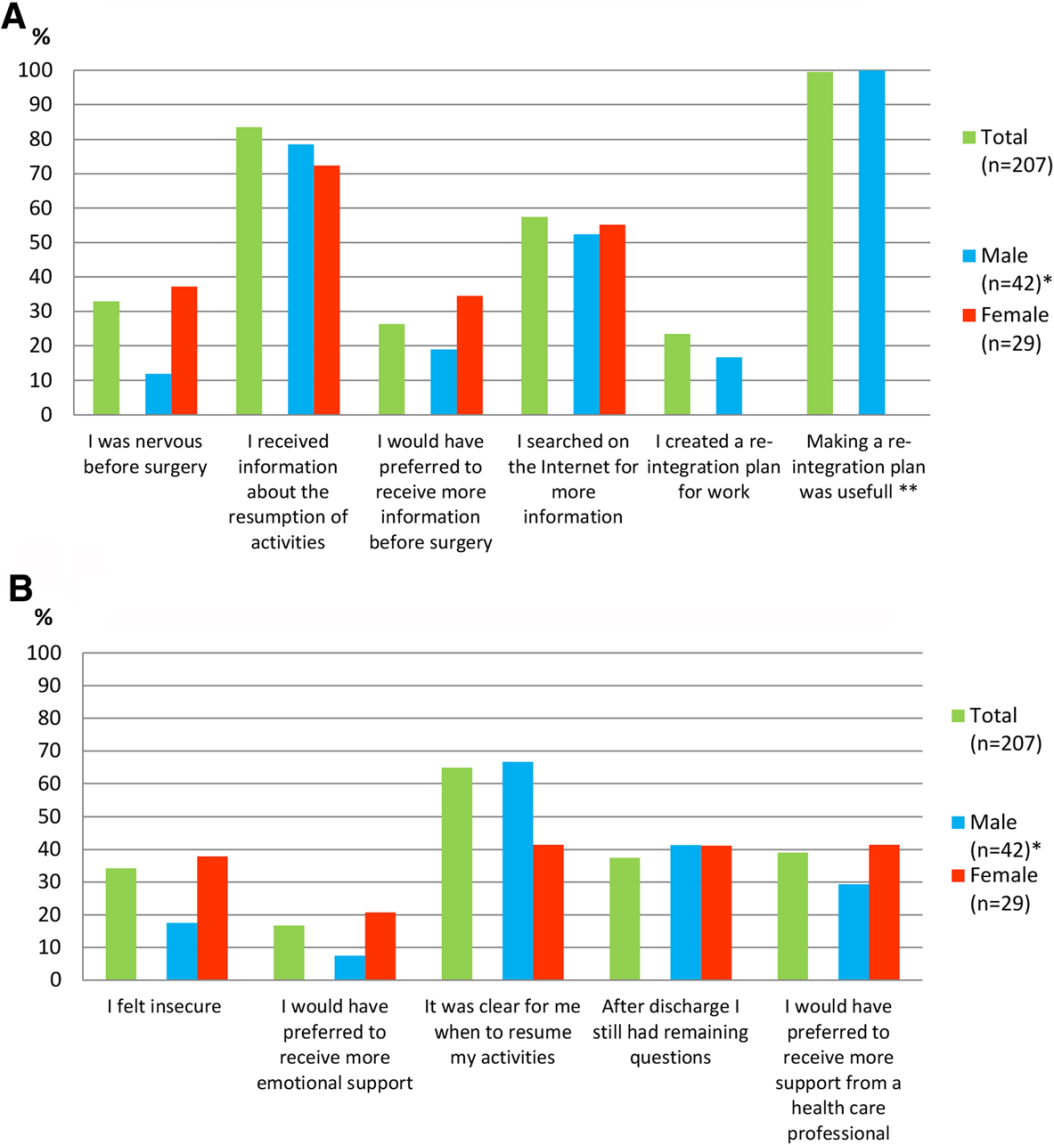
Patients’ statements. **a** Patients’ statements regarding the PRE-operative period **b** Patients’ statements regarding the POST-operative period. The bars present the percentage of the participants who agreed with the statement. * Differences between male and female evaluated in the group of patients who underwent a minor general surgical procedure (*n* = 71). ** Percentage of the 32 participants who created a re-integration plan

About one third of the participants (32.9% (68/207)) answered that they felt nervous before surgery. Compared to men, women were more likely to feel nervous (37.2% (11/29) vs 11.9% (5/42)) (Fig. 1).

##### Information supply

The majority of the participants (83.6%, 163/195) received information about the resumption of activities after surgery. The majority felt the information provided was sufficient, however, 26.3% (54/205) patients reported that they would have preferred to receive more information. This percentage was slightly higher in women compared to men (34.5% (10/29) vs 19.0% (8/42)). More than half of the participants (57.5% (115/200)) searched on the Internet for more information about the surgical procedure and recovery process.

##### Preparations with regard to return to work

Of the employed participants, 23.4% (32/137) reported that they made a plan regarding return to work (re-integration plan). 17 of them did this together with their employer and 15 did this on their own. In the subgroup of participants who underwent minor general surgical procedures, the creation of a re-integration plan was less common (0/20 of female participants and 4/24 male participants). All participants who made a reintegration plan except one, reported this to be useful and would do it again.

#### After surgery

Overall, 68.6% (142/203) of the participants reported that they were satisfied with their recovery period.

##### Mental health state

About one third of the participants (68/199) felt insecure during their recovery process. Women felt insecure more often than men (37.9% (11/29) vs 17.5% (7/40)). Thirty-four patients (16.7%) reported that they would have preferred more emotional or mental support after their surgical procedure. Women had a higher need for this than men (20.7% (6/29) vs 7.5% (3/40)).

##### Information supply

Confusion about the resumption of daily activities existed in about 35% of the patients (133/205). Recommendations regarding the resumption of activities provided by medical specialists, general practitioners (GP) and occupational physicians (OP), were reported to be inconsistent by 57% of the responders. The majority of patients (79.2%; 164/204) reported that they knew who they had to contact in case of physical complaints or questions. Seventy-six patients reported that they still had questions after discharge. The majority of these patients (76.3%; 58/76) did ask those questions, however only 59.6% (35/58) were satisfied after this contact.

##### Interaction with occupational physician (OP)

Of the employed participants, 27.0% (38/141) had at least one contact with their OP before or after surgery. Only 39.5% (15/38), designated this contact as useful.

##### Guidance during the recovery process

Of all participants, 39.0% (78/200) reported that they would have liked to receive more assistance by a health care professional during their recovery process. The mean time between surgery and the appointment in the outpatient clinic was four weeks. The timing of the postoperative appointment was adequate according to 76.2% of the patients. Around one in five patients (22.3%) preferred the appointment to be planned sooner. Only 1.6% would preferred the appointment to be later.

### Patients preferences regarding e-health

#### General

A total of 78.7% participants (155/197) agreed with the statement that there is a need for an e-health care program focusing on the deliverance of information and guidance during the perioperative period. Women were slightly more interested in this than men (88.9% compared to 73.2%). The majority of the patients (82.4%) stated that they were willing to spend about one to two hours of their time on such a program per week during the course of their recovery, while the other 17.6% were willing to spent even more than two hours per week.

#### Website

The majority of the patients (70.5%; 136/193) reported that if an e-health intervention (i.e. a specially developed website) had been available before or after their surgical procedure, they would have used it. This was slightly more the case in women compared to men (75.0%; 21/28 versus 62.2%; 23/37). Table 3 presents the functions patients reported to prefer on such a website, in order of popularity. Most items were assessed as useful by the majority of the participants; except two: the ability to give your employer or OP insight into a part of the website and a forum to talk with other patients. Most popular items were a page containing an overview of important telephone numbers, a list with frequently asked questions (FAQ) and the possibility to evaluate symptoms after surgery.

**Table 3:** Assessment of different website functions

Function	Useful	Not useful	No opinion	Number
Before surgery				
A practical list; what to manage before surgery?	157 (79.7%)	6 (3.0%)	34 (17.3%)	197
Information about the surgical procedure by text and animations	150 (76.1%)	11 (5.6%)	36 (18.3%)	197
Making a personal convalescence plan	141 (71.6%)	11 (5.6%)	45 (22.8%)	197
A video about the recovery process	132 (67.3%)	25 (12.8%)	39 (19.9%)	196
Making a reintegration plan for work	123 (62.4%)	14 (7.1%)	60 (30.5%)	197
A video about the surgical procedure	104 (52.5%)	39 (19.7%)	55 (27.8%)	198
After discharge				
Evaluation of symptoms	175 (88.8%)	5 (2.5%)	17 (8.6%)	197
Monitoring of recovery	141 (72.3%)	16 (8.2%)	38 (19.5%)	195
Focus on emotional well-being	117 (60.6%)	21 (10.9%)	55 28.6%	193
Inviting your GP to a part of the website	99 (50.8%)	40 (20.5%)	56 (28.7%)	195
Inviting your OP to a part of the website	64 (30.9%)	44 (21.3%)	87 (44.6%)	195
Inviting your employer to a part of the website	53 (27.2%)	62 (31.8%)	80 (41.0%)	195
General				
Contact details of involved health care professionals	178 (89.9%)	4 (2.0%)	16 (8.1%)	198
Frequently asked questions	160 (81.6%)	8 (4.1%)	28 (14.3%)	196
A list with frequently used medical terms	142 (72.8%)	9 (4.6%)	44 (22.6%)	195
Links to other websites	119 (64.0%)	15 (8.1%)	52 (28.0%)	186
Forum to chat with other patients	67 (32.4%)	56 (27.1%)	74 (32.6%)	197

#### Mobile phone application (app)

Almost half of the participants (48.2%; 95/197) reported that they would prefer to use the e-health care program by a mobile phone application as well. This was more often the case in men than in women (65.0%; 26/40 vs 48.3%; 14/29). Among the participants who reported that they are using the Internet on a smartphone or tablet in daily life (*n* = 168), only a slightly higher percentage (51.2%, 86/168) reported that they would prefer to use the e-health care program on a mobile phone application as well. Less than half of the patients (38.4%; 73/190) reported they would use the possibility to connect an activity tracker to their mobile phone application to track their activity during the recovery process.

#### E-consultation

Only a minority of the patients (17.6%; 35/199) would have preferred to replace their postoperative appointment in the outpatient clinic by electronic contact with their doctor (e-consult). This percentage increased slightly when only taking the participants into account who underwent minor surgery (27.9%; 19/68). The most reported reason for declining an e-consult was that the participants appreciated to have personal contact with their doctor (*n* = 153). However, the ability to use an e-consult to ask questions to a doctor or nurse during the recovery process in case of complaints, was assessed as useful by 57.6% (114/198) of the participants. One in five patients (21.2%; 42/198) assessed e-consultation as not being useful at all.

## Discussion

### Principal findings

In this survey study we analyzed the opinions of patients who underwent abdominal surgery about the availability of information and guidance they received before and after their surgical procedure. In addition, we evaluated their views on the use of e-health in the perioperative period. Although most participants reported that they had received some basic information about the surgical procedure and the recovery process, more than half of the participants searched the Internet for additional information. Most important reported shortcomings included the absence of detailed information about the resumption of (work) activities as well as the inconsistency between advice received by different healthcare professionals involved in the recovery process. A considerable proportion of patients (39%) reported that they would have liked to receive more assistance from a healthcare professional during their recovery process, and one in eight patients reported that they would have preferred more emotional support. Women had a slightly higher need for additional information and support than men.

A majority of participants expected an e-health program to be helpful during the recovery trajectory. A website was assessed as most useful. In particular practical functions focusing on the preparation for surgery and monitoring after surgery were expected to be valuable. There was less need for interaction with others (e.g. chat-function or forum, or giving other health care professionals access to the website). Also, the majority of patients opposed the option to replace the standard postoperative consult by an e-consult, since they preferred a personal contact with their surgeon.

### Comparison to the literature

When we compare our results to the qualitative study of Vonk Noordegraaf et al. which was at the base of the development of an e-health intervention for patients undergoing gynecological surgery, there are many similarities. In concordance to our own findings, Vonk Noordegraaf concluded that important shortcomings in current perioperative care were 1) the lack of instructions regarding the resumption of activities, 2) the inconsistency in the recommendations given by different healthcare providers and 3) the insecurity with respect to postoperative symptoms. However, there was inconsistency between the two studies on one point. In Vonk Noordegraaf’s study, participants reported that they would have preferred to have more contact with other patients during the perioperative course and subsequently suggested this to be one of the three most important tools to incorporate in the e-health intervention. In our study this option was rated as one of the three most unpopular items of a possible e-health intervention. Probably, the difference can be explained because of the difference in study population between the two studies. Another possible explanation could be the difference in study design between the two studies. The results from Vonk Noordegraaf’s study were derived from focus group discussions and therefor selection bias was highly likely because participants attending in this study were willing to discuss their problems with others. Finally, it could also be that there is indeed not a major need for it, which is in line with the low satisfaction rate with these functions in a previously tested e-health intervention for peri-operative care in gynecology [26].

Comparing our results to other recent publications, shows another inconsistency, namely the unpopularity of the postoperative appointment by an e-consult in our study [18–21, 27]. This difference might be explained by the fact that those previous studies mainly focused on the feasibility, safety and cost-effectiveness rather than the preferences of patients. Our study suggests, that even it would be feasible and safe from a medical perspective to replace the appointment in the outpatient clinic by an e-consult, from the Dutch patients’ perspective there is hardly any foundation for this. However, using e-consultations as an extra means of contact with the hospital in case of complaints, was rated as useful.

Earlier studies described differences in the recovery process after cardiac surgery between men and women [28–34]. These studies conclude that during the recovery process women suffered from more symptoms, showed lower functioning scores and had a higher re-admission rate than men, which could not be explained because of illness severity or other patient characteristics [29, 30, 32, 33]. When specifically focusing on gender differences according to the effectivity of e-health interventions applied in the recovery process after cardiac surgery, data trends in one study showed that the intervention had greater impact on women than on men in the postoperative course [34]. We only detected some minor differences according to gender: overall women showed a slight higher need to information, extra support or e-health compared to men. However, the results regarding this topic should be interpreted with caution; although we selected the most homogeneous group possible within the limits set by this study for comparing men and women, the remaining group was small, age differed significantly and the type of minor surgical procedures differed between men and women.

### Strengths and limitations

A strength of this study lies in the extensiveness of the questionnaire and the fact that the questionnaire was developed based on the results of a qualitative study. We approached all patients who underwent all types of surgical abdominal procedures over the period of one year, which has led to a good clinical representation.

However, this study has also limitations. First, the recruitment of patients was limited to an academic hospital. This may have influenced the results because, in general, in academic hospitals the more complicated surgical procedures are being performed. Nonetheless, the indication for surgery in our study population was in most cases benign and the complication rate was moderate. In addition, perioperative care provided in the academic and non-academic hospitals in the Netherlands is quite similar; based on the guidelines of the Dutch Society of Obstetrics and Gynecologists (NVOG), patients get verbal instructions by a nurse or physician at discharge and will receive a leaflet with some recovery instructions. [9, 35]. Moreover, patients receive an appointment at the outpatient clinic between two and six weeks after surgery. Therefore we assume, that the results are generalizable. Second, because of the retrospective design of this study the time between surgery and the questionnaire varied between 5 weeks and 62 weeks between the study participants. This might have resulted in recall bias as well as in difference between pre-surgery and post-surgery answers. For example, if patients underwent surgery without complications they would be more likely to answer that they had no need for extra information or support than when they were questioned before surgery. However, since the complication rate was normal in this study, we think that this only could have led to an underestimation regarding the need for information and support. A third limitation might be the relative low response rate (57.2%). However, we were able to compare baseline characteristics between participants and non-responders. Responders were significantly older (46.59 vs 39.51), which may have influenced the results. Possibly, patients’ needs and preferences regarding e-health were underestimated, since older adults generally make less use of new technologies [36]. In addition, the responders underwent gynecological procedures more frequently in comparison to the non-responders, however, the ratio gynecological procedures versus general surgical procedures was equal in the groups of responders. Although we were able to perform a non-response analysis regarding some important baseline characteristics, we could not rule out that there were other important differences between the two groups which we were not able to compare. For example Internet use: 95.1% of the study participants reported that they are using the Internet several times a week or on a daily basis. We do not have data regarding this topic from the non-participants. So it is therefore possible that the rate of Internet use was much lower in this group, which makes the generalizability of the results, mainly regarding the preferences regarding e-health, lower. Finally, the heterogeneity in terms of the many types of surgical procedures included in this study, could also be pointed as a limitation. However, we had a good rationale for this since we aimed to evaluate whether the results obtained with a qualitative study in a gynecological population, were also applicable to a broader population.

## Conclusions

The results of this study showed that most important shortcomings in current perioperative care in patients undergoing abdominal surgery are the lack of detailed advice about the resumption of activities following surgery and the limited guidance of professionals during the recovery process. E-health is expected to be very useful tool to overcome these shortcomings. The results of this study can be used by health care professionals and policymakers when developing these type of e-health interventions for perioperative care. It provides a broad overview of the different phases of perioperative care and the generalizability of the study is high. Future research should include a cost-effectiveness evaluation including a process evaluation of such e-health interventions to evaluate the feasibility. In addition, future research should focus on gender differences in postoperative recovery, since trends of this study suggest that there may be differences.

## Abbreviations

ASA: American society of anesthesiologists classification
BMI: Body mass index
FAQ: Frequently asked questions
GP: General practitioner
LS: Laparoscopic procedure
O: Open procedure
OP: Occupational physician
SES: Social Economic Status
